# Infectious Chikungunya Virus in the Saliva of Mice, Monkeys and Humans

**DOI:** 10.1371/journal.pone.0139481

**Published:** 2015-10-08

**Authors:** Joy Gardner, Penny A. Rudd, Natalie A. Prow, Essia Belarbi, Pierre Roques, Thibaut Larcher, Lionel Gresh, Angel Balmaseda, Eva Harris, Wayne A. Schroder, Andreas Suhrbier

**Affiliations:** 1 Inflammation Biology Laboratory, QIMR Berghofer Medical Research Institute, and the Australian Infectious Diseases Research Centre, Brisbane, Queensland, Australia; 2 School of Chemistry and Molecular Biosciences, University of Queensland, Brisbane, Queensland, Australia; 3 CEA, iMETI, Division of Immuno-Virology, Université Paris Sud and Center for immunology of viral infections and autoimmune diseases Inserm, UMR 1184, Fontenay-aux-Roses, France; 4 Institut National de Recherche Agronomique, Nantes, France; 5 Sustainable Sciences Institute, Managua, Nicaragua; 6 Laboratorio Nacional de Virología, Centro Nacional de Diagnóstico y Referencia, Ministry of Health, Managua, Nicaragua; 7 Division of Infectious Diseases and Vaccinology, School of Public Health, University of California Berkeley, Berkeley, CA, United States of America; Singapore Immunology Network, Agency for Science, Technology and Research (A*STAR), SINGAPORE

## Abstract

Chikungunya virus (CHIKV) is a reemerging, ordinarily mosquito-transmitted, alphavirus that occasionally produces hemorrhagic manifestations, such as nose bleed and bleeding gums, in human patients. Interferon response factor 3 and 7 deficient (IRF3/7^-/-^) mice, which are deficient for interferon α/β responses, reliably develop hemorrhagic manifestations after CHIKV infection. Here we show that infectious virus was present in the oral cavity of CHIKV infected IRF3/7^-/-^ mice, likely due to hemorrhagic lesions in the olfactory epithelium that allow egress of infected blood into the nasal, and subsequently, oral cavities. In addition, IRF3/7^-/-^ mice were more susceptible to infection with CHIKV via intranasal and oral routes, with IRF3/7^-/-^ mice also able to transmit virus mouse-to-mouse without an arthropod vector. Cynomolgus macaques often show bleeding gums after CHIKV infection, and analysis of saliva from several infected monkeys also revealed the presence of viral RNA and infectious virus. Furthermore, saliva samples collected from several acute CHIKV patients with hemorrhagic manifestations were found to contain viral RNA and infectious virus. Oral fluids can therefore be infectious during acute CHIKV infections, likely due to hemorrhagic manifestations in the oral/nasal cavities.

## Introduction

Chikungunya virus (CHIKV) is a member of a group of globally distributed, arthritogenic alphaviruses that are ordinarily transmitted by mosquitoes. The largest documented outbreak of CHIKV disease ever recorded began in 2004 and has resulted in an estimated 1.4–6.5 million cases, mainly in Africa and Asia, with imported cases reported in over 40 countries [1,2]. The outbreak has recently caused thousands of cases in Papua New Guinea [3], the Caribbean [4] and Central America, with autochthonous transmission also reported in the USA [1,5].

We recently reported a mouse model of CHIKV-induced hemorrhagic fever and hypovolemic shock using interferon response factor 3 and 7 deficient (IRF3/7^-/-^) mice [6]. The study illustrated that deficiencies in type I interferon responses were sufficient to elicit hemorrhagic manifestations after CHIKV infection [6]. In a non-human primate model of CHIKV using cynomolgus macaques, we previously observed gingival bleeding in 50% of infected monkeys [7]. Although the primary disease manifestations of CHIKV infections in humans are acute fever and rash, and acute and chronic polyarthralgia/polyarthritis, hemorrhagic manifestations are occasionally seen (in ≈2–4% of patients) in both infected children [8,9,10,11] and adults [12,13,14,15]. Clinical signs of hemorrhage in CHIKV patients include purpura (bruising), hematemesis (vomiting blood), melena (pathognomonic of upper gastrointestinal bleeding), epistaxis (nose bleed) [12,16], petechiae (small red or purple spots on the skin), hematuria (blood in urine) [17], and gingivorrhagia (bleeding gums) [15]. The latter was reported in ≈1% of adults [15]; however, in CHIKV-infected dental patients, an Indian study showed the incidence can be much higher (>50%) [18]. Herein, using data from mice, monkeys and humans, we show that infectious CHIKV can be present in oral fluids/saliva, with hemorrhagic manifestations in the oral and/or nasal cavities during the viremic period likely responsible.

## Materials and Methods

### Animal ethics statement

All mouse work was conducted in accordance with good animal practice as defined by the National Health and Medical Research Council of Australia. Mouse work was approved by the QIMR Berghofer Medical Research Institute animal ethics committee and was conducted in biosafety level-3 facility at the QIMR Berghofer. To limit suffering all infected IRF3/7^-/-^ mice were euthanized (carbon dioxide asphyxiation) before hemorrhagic symptoms became severe [6].

Monkey samples were obtained from monkeys enrolled in previously described studies [7,19]; no monkeys were specifically used for the studies described herein in accordance with Directive 2010/63/CE (article 18 *sharing tissues and organs*). Monkey studies were approved by the regional animal care and use committee (“Comite Regional d’Ethique sur l’experimentation animale Ile de France Sud,” Fontenay-aux-Roses, France) in accordance with European directive 86/609/EEC. Monkeys were housed in a BSL3 facility at the Centre d’Energie Atomique (CEA) (Fontenay-aux-Roses, 92265 France) (Permit number A 92-032-02), in accordance with Office for Laboratory Animal Welfare (OLAW, USA; #A5826-01) standards at the CEA in accordance with the French national regulation under the number B-92-032-02 for animal use and 2005–69 for breeding. The monkeys were housed individually in stainless-steel squeeze-back cages (cage size following the directive 2010/63/CE and French law “décret 2013–118 from February 1^st^ 2013”) in a climate-controlled room, 21 ± 1°C, and relative humidity 55 ± 5% with a 12 h light/dark cycle. The monkeys were fed daily, *ad libitum*, with specialized food and fruits. Enrichments, like toys and special “hidden” foods, were provided. The animals were sedated with ketamine chlorhydrate (Rhone-Merieux, Lyon, France) for virus injection, and blood and saliva collection as described [19].

### Mouse infections and virus titrations

IRF3/7^-/-^mice were kindly provided by M. S. Diamond (Washington University School of Medicine, St. Louis, MO); these and Rag1^-/-^ mice were bred at the QIMR Berghofer animal house facility. C57BL/6 mice were purchased from Animal Resources Center (Canning Vale, WA, Australia).

Donor mice were infected with the CHIKV isolate LR2006-OPY1 (2x10^4^ CCID_50_), grown in C6/36 cells [20] (GenBank KT449801) and shown to be mycoplasma free [21], via i.p. inoculation in 50 μl of medium (RPMI 1640 supplemented with 2% endotoxin free [22] fetal calf serum). For mucosal infection studies, CHIKV was applied by gently applying 40 μl of CHIKV-containing medium (2x10^4^ CCID_50_) via pipette into the nose or mouth of scruff-restrained mice.

Mouth washings (from the oral cavity) were obtained by gently rinsing the oral cavity of scruff-restrained mice with 100 μl of medium (2% fetal calf serum), aspirating the washings back into the pipette and repeating this 3 times, taking care not to damage the mucosa with the pipette tip. Vaginal washings were obtained by gently rinsing the vagina with 50 μl of medium and aspirating the washings back into the pipette and repeating this 3 times, again taking care not to damage the mucosa. Urine was collected after scruff-induced urination [6]. Infectious virus titers in the washings/urine were determined as described [6].

### Mouse-to-mouse transmission

Infected (donor) mice were co-housed with uninfected (recipient) mice as indicated. Infection of donor IRF3/7^-/-^ mice was confirmed by clinical symptoms (hunching, ruffled fur, foot swelling) [6] and donor C57BL/6 mice by IgG ELISA [23]. Infection of recipient IRF3/7^-/-^ mice was indicated by clinical symptoms [6] and/or RT-PCR [24]. Infection of recipient C57BL/6 mice was tested by IgG ELISA [23].

### Monkey infections and viral load assessments

Monkeys were enrolled in experiments described previously [7,19]. Briefly, captive-bred 3–5 year old cynomolgus macaques (*Macaca fascicularis*) were infected with either 10^6^ PFU (equivalent to 10^5^ 50% animal infectious doses) or 10^3^ PFU (100 50% animal infectious doses) of CHIKV isolate LR2006-OPY1 via inoculation (1 ml) into the saphenous vein. Plasma and saliva viral RNA copy numbers were determined using quantitative real time RT-PCR, and infectious viral titers were determined by CCID_50_ assay using BHK-21 cells and/or PFU using Vero cells, as described [7]. A dose of 10^8^ CCID_50_/ml is equivalent to 1.8 × 10^8^ PFU/ml and 1.8 ± SD 0.9 × 10^10^ viral RNA copies/ml. Saliva (0.2–2ml) was collected during routine clinical examinations after ketamine anesthesia using plastic pipettes inserted into the oral cavity between cheek and gum. Samples were examined for color (bleeding), were centrifuged and stored at -80°C.

### Analysis of saliva samples from human CHIKV patients

From August 2014 to June 2015, saliva samples were obtained from 13 CHIKV positive patients (who were dengue negative) presenting with hemorrhagic manifestations; 5 patients were enrolled via a cohort study and 8 patients were enrolled via a hospital-based study in Nicaragua (see below). A patient was considered positive for DENV infection when laboratory tests met one or more of the following criteria: (i) dengue viral RNA was detected by RT-PCR [25,26]; (ii) DENV was isolated [25]; (iii) seroconversion of DENV-specific IgM was detected by MAC-ELISA in paired acute and convalescent samples [25,27]; and/or (iv) DENV-specific antibody titer by inhibition ELISA [28,29,30] demonstrated a 4-fold or greater increase between acute and convalescent sera. CHIKV infection was determined by RT-PCR testing of blood samples (see below). Saliva was collected by spitting into a tube, transported at 4–8°C and was aliquoted and stored at -80°C. Samples were thawed and RNA extracted as described [31]. The presence of CHIKV mRNA was determined by a TaqMan real-time RT-PCR [32] using primer/probe sets designed to detect Asian and ECSA genotypes of CHIKV (Lanciotti RS, personal communication): primers, forward (CHIKV 3855) 5′-GAGCATACGGTTACGCAGATAG-3′, reverse (CHIKV 3957-Asian) 5′-TACTGGTGATACATGGTGGTTTC-3′ and (CHIKV 3957-ECSA) 5′-TGCTGGTGACACATGGTGGTTTC-3′; probes, (CHIKV 3886 FAM-Asian) 5′-FAM-ACGAGTAATCTGCGTACTGGGACGTA-BHQ1-3′ and (CHIKV 3886 FAM-ECSA) 5′-FAM-ACGAGTCATCTGCGTATTGGGACGCA-BHQ1-3′. Crossing threshold values for positive were <38 according to protocols supplied by the Centers for Disease Control and Prevention, USA [32]. Each saliva RNA sample was tested independently 3 times with appropriate positive and negative controls. Virus isolation was undertaken by incubating saliva with Vero cells (ATCC CCL-81), and wells showing cytopathic effects were then analyzed by RT-PCR as above.

The Pediatric Dengue Cohort Study is an ongoing community-based prospective study established in 2004 in Managua, Nicaragua [33,34]. The study is based at the local municipal health center, the Health Center Sócrates Flores Vivas (HCSFV). The area served by the HCSFV is District II of Managua, a low- to middle-income area with a population of approximately 62,500. Children aged two to nine years old living in District II were enrolled, and new participants are enrolled each year. Children are withdrawn from the study when they reach 15 years of age. Participants are encouraged to present at the first sign of illness to the HCSFV, where study physicians provide medical care and screen for signs and symptoms of dengue, and since August 2014, of CHIKV infection.

An ongoing hospital-based prospective study of dengue was established in 1998 in the Infectious Disease Ward of the Hospital Infantil Manuel de Jesús Rivera Hospital (HIMJR), the national pediatric reference hospital in Managua [35,36,37]. In-patients and out-patients between 6 months and 14 years of age were enrolled when they present at the HIMJR with documented or reported fever of less than 7 days and one or more of the following signs and symptoms: headache, arthralgia, myalgia, retro-orbital pain, positive tourniquet test, petechiae or others signs of bleeding. Enrollment occurs each year during August-January, the peak dengue and CHIKV season. Children with a diagnosis other than dengue or CHIKV and children weighing less than 8 kg were excluded. Upon enrollment, a medical history was taken, and a complete physical exam performed.

The protocols for the Pediatric Dengue Cohort Study and the hospital-based study were reviewed and approved by the Institutional Review Boards of the University of California, Berkeley, and of the Nicaraguan Ministry of Health. Parents or legal guardians of all patients in both studies provided written informed consent, and patients ≥6 years of age provided assent.

## Results

### Hemorrhage and infectious virus in the oral cavity of CHIKV infected IRF3/7^-/-^ mice

Hemorrhagic manifestations are generally not observed in CHIKV infected wild-type C57BL/6 mice. In contrast, we have previously shown that IRF3/7^-/-^ mice infected s.c. in the foot with CHIKV (i) had high viraemias, ≈10^7^−10^10^ CCID_50_/ml, about 4 logs higher than in wild-type mice between days 3–5 post infection, (ii) showed hemorrhagic symptoms (i.e. edematous foot swelling, hypothermia, oliguria) [6] and (iii) developed hypovolemic shock requiring euthanasia [6]. When IRF3/7^-/-^ mice where inoculated i.p., mortality, foot swelling, viremia and histological signs of hemorrhage were similar (Fig A in S1 File) to that reported for s.c. inoculation in these mice [6], with the i.p. route of inoculation chosen to avoid the possibility that mice may become infected orally by licking some viral inoculate (potentially exuding due to back-pressure) from the s.c. injection sites.

**Fig 1:**
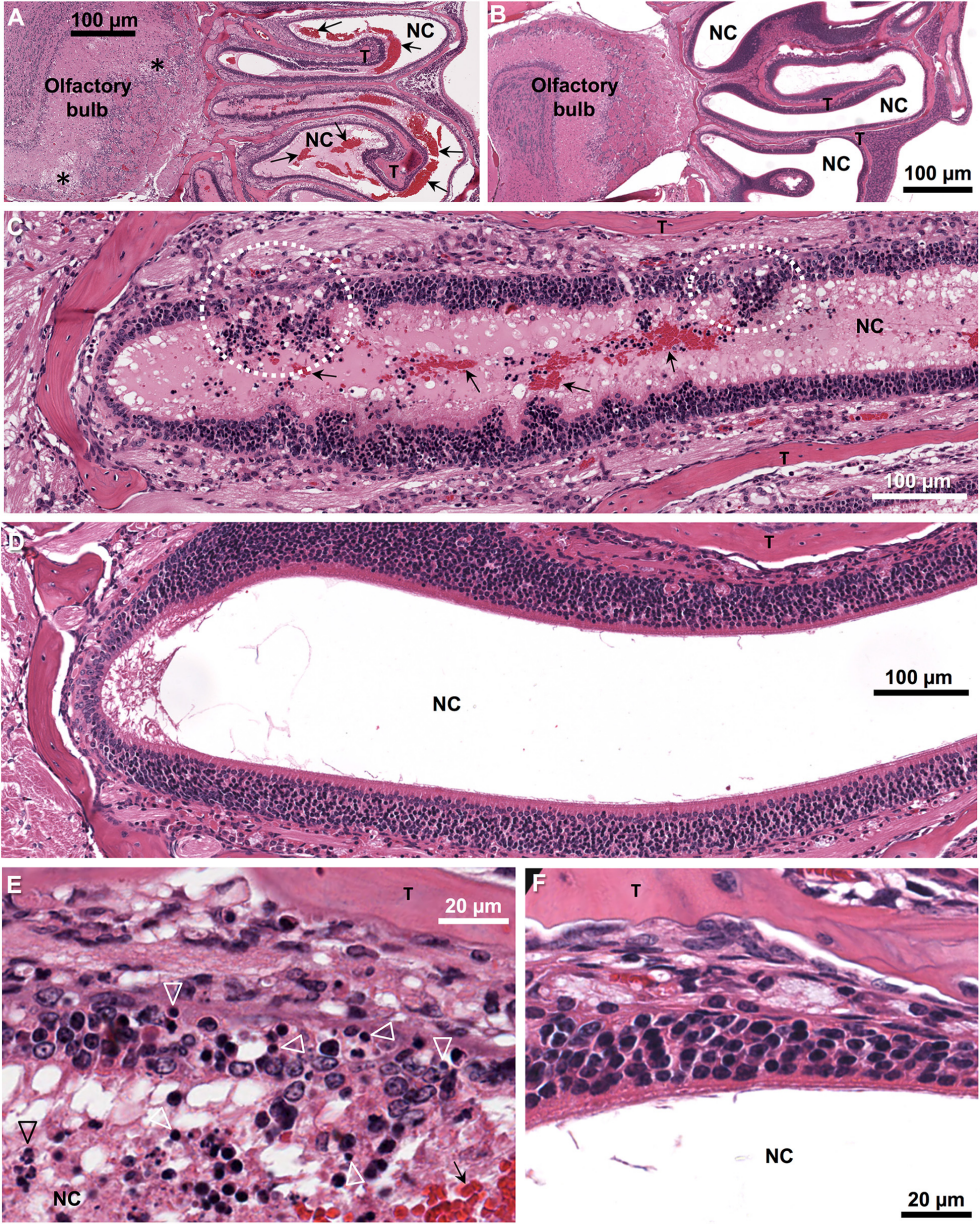
Hemorrhagic lesions in the olfactory epithelium of CHIKV infected IRF3/7^-/-^ mice. (A, C, E) H&E of the head of IRF3/7^-/-^ mouse day 5 post CHIKV infection and (B, D, F) H&E of the head of a control uninfected IRF3/7^-/-^ mouse; T—turbinate bones, arrows—red blood cells, NC—nasal cavity. (A) Low magnification image showing extensive red blood cells and mucous exudates (pink staining material) in the nasal cavities. (*—large foci of vacuolization in the olfactory bulb). (B) The same region and magnification as in A for an uninfected mouse. (C) Medium magnification image showing breaches in the olfactory epithelium (dotted white circles). (D) The same region and magnification as in C for an uninfected mouse. (E) High magnification image of a lesion in the olfactory epithelium showing pyknotic nuclei (white arrow heads) and some neutrophils (black arrow head). (F) The same region and magnification as in E for an uninfected mouse.

Histological analysis of the heads of IRF3/7^-/-^ mice at day 5 after i.p. infection showed that significant amounts of red blood cells were clearly evident in nasal cavities (Fig 1A, compare with uninfected control Fig 1B). The nasal cavities drain into the oral cavity, where red blood cells were also present (data not shown). Lesions disrupting the integrity of the olfactory epithelium of the turbinates were clearly evident in these mice (Fig 1C, compare with Fig 1D), with these lesions containing pyknotic cells and neutrophils (Fig 1E, compare with Fig 1F). No lesions were seen in the oral mucosa or the respiratory epithelium of IRF3/7^-/-^ mice (data not shown). In infected C57BL/6 mice, only occasionally were low levels of red blood cells observed in nasal cavities, with no overt disruptive lesions in the epithelium evident (Fig B in S1 File). These results suggest that disruptive lesions in the olfactory epithelium lead to significant levels of blood entering the nasal, and subsequently, the oral cavity in CHIKV infected IRF3/7^-/-^ mice.

**Fig 2:**
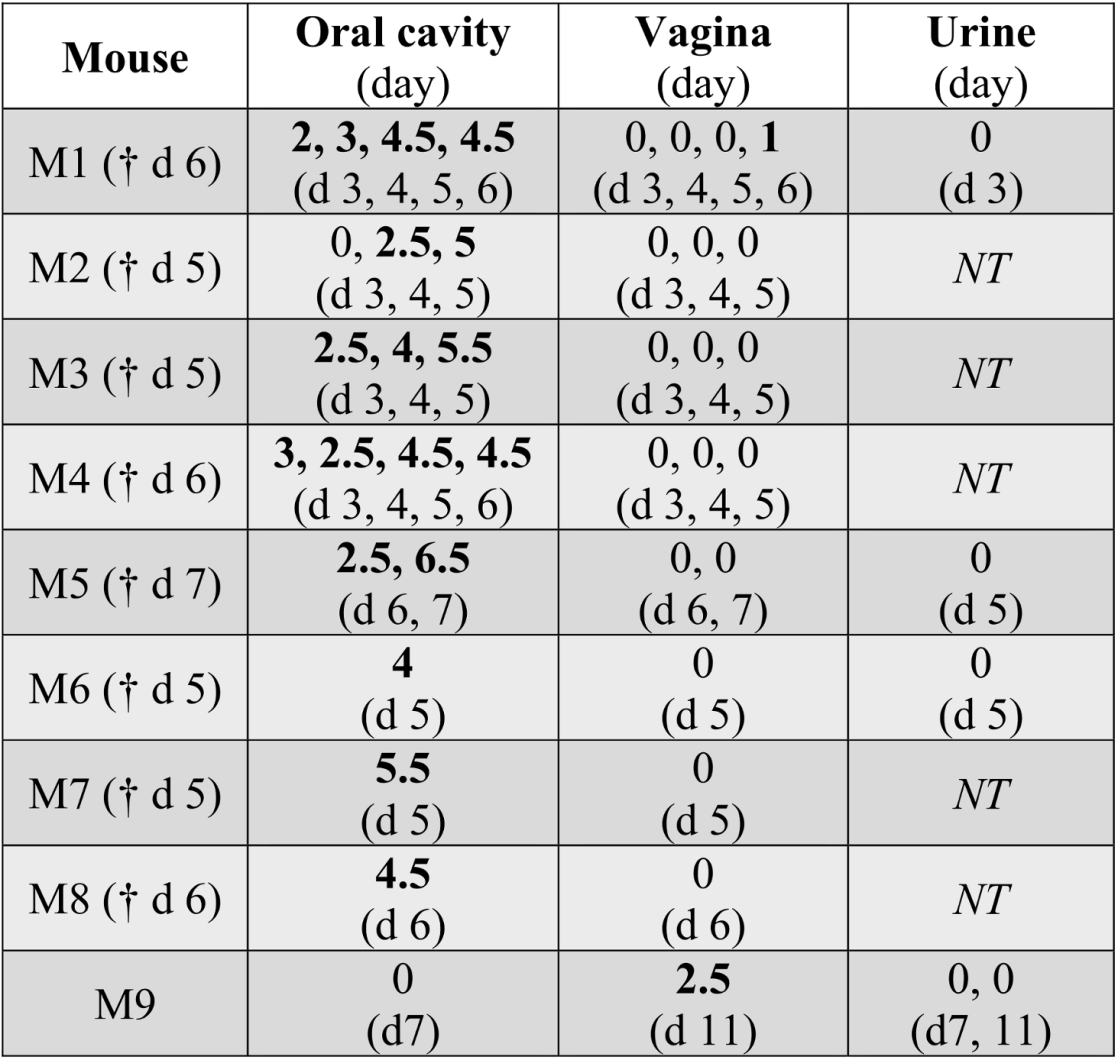
Virus in oral cavity washings, vaginal washings and urine of CHIKV-infected IRF3/7^-/-^ mice. Mice were infected with CHIKV i.p. (n = 9) and when clinical symptoms of hypovolemic shock became apparent (indicating onset of hemorrhagic manifestations) [6], mouth washings (Oral cavity), vaginal washings (Vagina) and urine (where possible), were collected and viral titers determined and expressed as log_10_CCID_50_/ml of washing medium on the indicated day (which is indicated in brackets below the titer Figs, respectively). † day when mouse was euthanized. *NT*–not tested.

Infectious virus could be reliably recovered from the oral cavity (via mouth washings) of CHIKV-infected IRF3/7^-/-^ mice between days 3 and 6 (Fig 2). Vaginal washings from IRF3/7^-/-^ mice were only occasionally positive (Fig 2); (males and females were housed separately). Urine from IRF3/7^-/-^ mice was negative in all samples tested (Fig 2); however, hemorrhage-induced oliguria [6] meant urine samples were difficult to obtain during the peak of hemorrhagic disease.

Infectious virus was not recovered from mouth or vaginal washings of 6 viraemic C57BL/6 mice, tested on days 1, 2, 3, 4, and 8 post infection (data not shown). Urine was also negative; 15 samples were tested (from the same mice days 1 to 4) (data not shown). Rag1^-/-^ mice (n = 5) infected with CHIKV s.c. were also tested, with no infectious virus found in mouth washings, although one mouse had a CHIKV-positive vaginal washing (data not shown). Rag1^-/-^ mice are unable to clear a CHIKV infection and show a persistent steady-state viraemia [38], but adult mice show no overt disease nor hemorrhagic pathology [24].

### Infection of mice with CHIKV via mucosal surfaces

**Fig 3:**
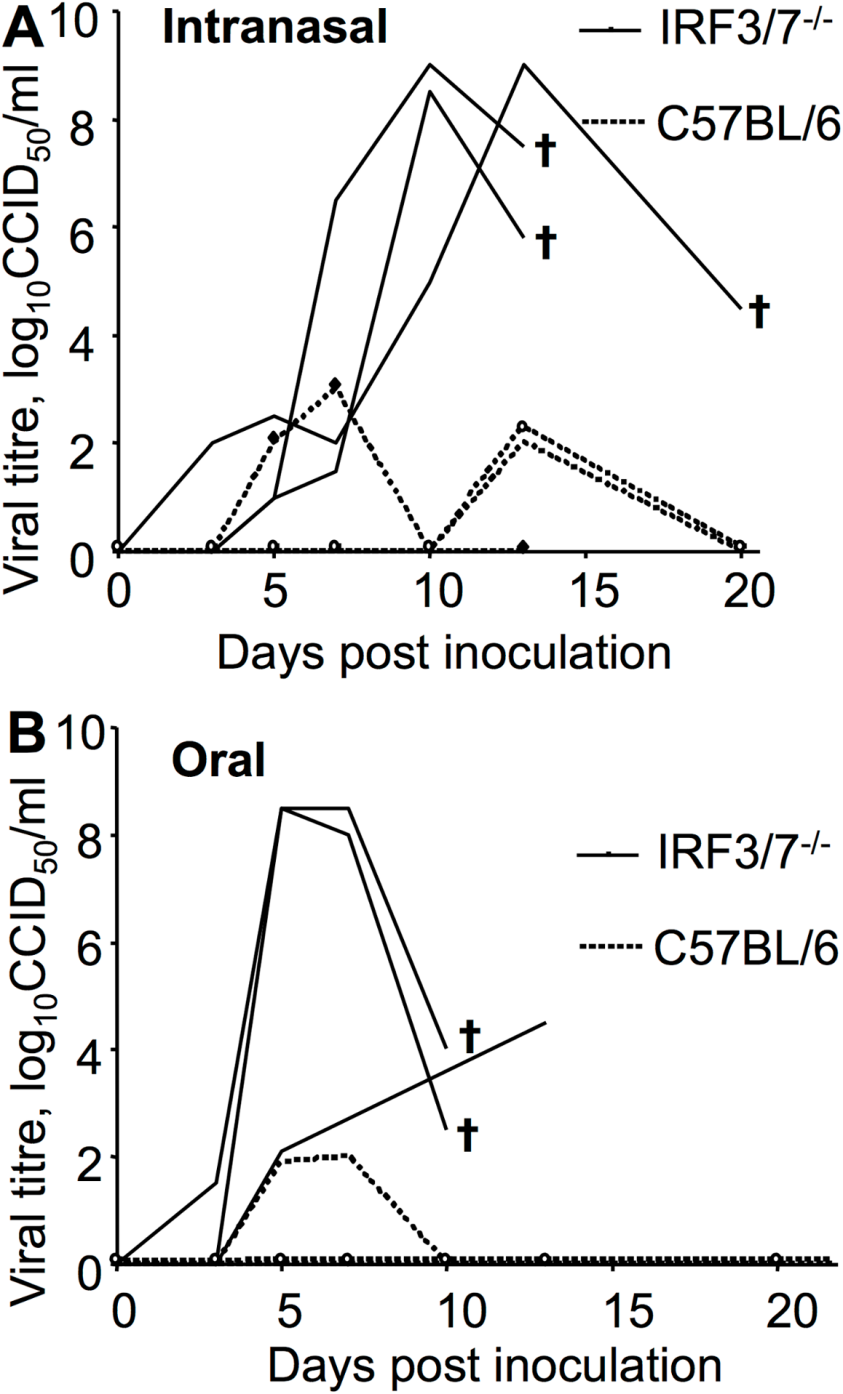
CHIKV infection via intranasal and oral routes. CHIKV was inoculated via pipette into the nose or mouth of restrained IRF3/7^-/-^ and C57BL/6 mice (n = 3 per group, 12 mice total). Viraemia was monitored as described, with 2 log_10_CCID_50_ the limit of detection [6]. †—mouse euthanized.

Inoculation of IRF3/7^-/-^ and C57BL/6 mice (with 2 x 10^4^ CCID_50_ of virus) by intranasal and oral routes showed that the IFNα/β response-deficient IRF3/7^-/-^ mice are more susceptible to infection via mucosal surfaces than C57BL/6 mice, with viraemias not detected or at the limit of detection (2 log_10_CCID_50_) in C57BL/6 mice (Fig 3). Intranasal infection of BALB/c mice has previously been reported [39]. (It might be noted that intranasal and oral inoculations may, in part, target the same tissue sites).

Vaginal inoculations of IRF3/7^-/-^ and C57BL/6 mice (with 2 x 10^4^ CCID_50_ of virus) did not result in detectable serum viraemias in either mouse strain, although using a higher dose of virus (1.5 x10^5^ CCID_50_), IRF3/7^-/-^ mice could be infected via the vaginal route (data not shown).

### Mouse-to-mouse transmission of CHIKV

As infectious virus could be recovered from oral cavities and mice could be infected via oral and intranasal routes, we sought to determine whether mouse-to-mouse transmission of CHIKV was possible in IRF3/7^-/-^ mice. Mice were infected with CHIKV and were used as “Donor” mice. These Donor mice were housed together with naïve (uninfected) IRF3/7^-/-^ mice referred to as “Recipient” mice). Mouse-to-mouse transmission, with Recipient mice becoming infected, was repeatedly observed for female mice; less so for male mice (Table 1). The reason for this gender difference is unclear, but may be due to gender differences in social behavioral [40].

**Table 1:** Mouse-to-mouse transmission of CHIKV. Donor mice were infected with CHIKV and were then housed in the same cage as naïve Recipient mice. Infection of Recipient IRF3/7^-/-^ mice become apparent at the indicated times (Column 3) by onset of clinical symptoms (hunching, ruffled fur, swollen feet) indicating onset of CHIKV-induced hemorrhagic shock [6]. Samples for viraemia and/or RT-PCR were taken on the days indicated in Column 3.

No. infected (Donor)	No. uninfected (Recipient)	Recipient mice with clinical symptoms	Evidence of recipient infection
3 IRF3/7^-/-^ F	1 IRF3/7^-/-^ F	1 mouse day 15	Viraemia ^1^ & RT-PCR +ve. Both feet swollen. Euthanized
2 IRF3/7^-/-^ F	2 IRF3/7^-/-^ F	1 mouse day 10	Both feet swollen. Euthanized
2 IRF3/7^-/-^ F	3 IRF3/7^-/-^ F	1 mouse day 11	Both feet swollen. Euthanized
2 IRF3/7^-/-^ M	2 IRF3/7^-/-^ M	1 mouse day 13	RT-PCR +ve. Euthanized
1 IRF3/7^-/-^ M	4 IRF3/7^-/-^ M	0	None ^2^
3 IRF3/7^-/-^ M	1 IRF3/7^-/-^ M	0	None ^2^
2 C57BL/6 F	2 IRF3/7^-/-^ F	0	None ^2^
3 IRF3/7^-/-^ F	2 C57BL/6 F	0	None ^3^
3 IRF3/7^-/-^ F	2 C57BL/6 F	0	None ^3^
**Mesh divider**			
3 IRF3/7^-/-^ F	3 IRF3/7^-/-^ F	0	None
3 IRF3/7^-/-^ F	3 IRF3/7^-/-^ F	0	None
3 IRF3/7^-/-^ M	3 IRF3/7^-/-^ M	0	None

^1^ 6.5 log_10_CCID_50_/ml.

^2^ As assessed by clinical symptoms.

^3^ Infection of Recipient C57BL/6 mice was determined by IgG ELISA 17 days after the last Donor IRF3/7 mouse was euthanized. In a separate series of experiments, a mesh divider was placed between Donor and Recipient mice in the same cage (Mesh divider), allowing air circulation, but not physical contact, between Donor and Recipient mice.

No transmission was observed between IRF3/7^-/-^ and C57BL/6 mice (Table 1). Although these experiments are hardly exhaustive, the data is consistent with the notion that both haemorrhage in the donor (resulting in infectious virus in the mouth) and high susceptibility in the recipient (Fig 3), promotes mouse-to-mouse transmission.

Transmission was not seen when a mesh divider was placed between the “Donor” and “Recipient” mice (Table 1), suggesting aerosol transmission was not involved.

### Infectious virus in the saliva of CHIKV-infected monkeys

To extend the observations from the IRF3/7^-/-^ mouse model to a more relevant [41] primate model, the presence of viral RNA and infectious virus was assessed in the saliva of CHIKV-infected non-human primates (cynomolgus macaques), as we have previously observed gingival bleeding in half the infected animals in this primate model of CHIKV infection and disease [7]. Saliva samples were available for 6 CHIKV-infected monkeys enrolled in previous studies [7]. CHIKV RNA was detected in saliva on or near the peak viraemia (Table 2; Fig C in S1 File), with infectious virus recovered from the saliva of 4 animals on or near the peak viraemia (Table 2).

**Table 2:** Presence of CHIKV RNA and infectious CHIKV in the saliva of CHIKV infected cynomolgus macaques. Monkeys A and B were infected with 10^6^ PFU and monkeys C-F with 4 x 10^3^ PFU of CHIKV. Viral RNA copies were determined by quantitative real time RT-PCR; (the full data set for animals C-F is graphed in Fig C in S1 File. Infectious virus titers were determined by CCID_50_ assay using BHK-21 cells or PFU assay using Vero cells. Day 2 usually represents peak viraemia [16]. ND—not detected. *NT*—not tested. ^1^ Blood noted in sample. Where PFU and CCID_50_ values were determined, tests were performed at different times with different storage periods.

Day post infection (monkey)	Plasma, CHIKV RNA copies/ml	Saliva, CHIKVRNA copies/ml	Saliva, infectious virus titer/ml
0 (A)	ND	ND	ND
1 (A)	1.18 x 10^9^	7 x 10^2^	ND
2 (A)	4.66 x 10^9^	ND	ND
0 (B)	ND	ND	ND
1 (B)	1.78 x 10^8^	6 x 10^3^	**10** ^**3**^ **CCID**_**50**_
2 (B)	3.92 x 10^8^	6 x 10^2^	ND
0 (C)	ND	ND	*NT*
1 (C)	2.11 x 10^7^	1.38 x 10^4^	*NT*
2 (C)	8.51 x 10^8^	1.10 x 10^6^	**1.8 x 10** ^**3**^ **PFU** ^**1**^
4 (C)	6.07 x 10^6^	ND	ND
5 (C)	2.23 x 10^4^	ND	ND
7 (C)	2.02 x 10^3^	ND	ND
0 (D)	ND	ND	*NT*
1 (D)	3.22 x 10^7^	7.74 x 10^2^	*NT*
2 (D)	7.92 x 10^8^	ND	**30 PFU and 10** ^**2**^ **CCID**_**50**_
4 (D)	1.02 x 10^6^	3.09 x 10^2^	*NT*
5 (D)	1.55 x 10^5^	*NT*	*NT*
7 (D)	4.4 x 10^2^	ND	*NT*
0 (E)	ND	*NT*	*NT*
1 (E)	4.16 x 10^7^	*NT*	*NT*
2 (E)	1.55 x 10^9^	1.27 x 10^5^	*NT*
4 (E)	4.75 x 10^7^	**ND**	*NT*
5 (E)	6.63 x 10^6^	2.63 x 10^3^	**ND**
7 (E)	6.86 x 10^3^	*NT*	*NT*
0 (F)	ND	*NT*	*NT*
1 (F)	1.3 x 10^7^	ND	*NT*
2 (F)	5.17 x 10^9^	1.6 x 10^6^	**2.2 x 10** ^**3**^ **PFU and 1.3 x 10** ^**3**^ **CCID**_**50**_
4 (F)	8.27 x 10^7^	5.95 x 10^4^	*NT*
5 (F)	2.97 x 10^6^	8.07 x 10^2^	**ND**
7 (F)	2.16 x 10^4^	2.4 x 10^3^	*NT*

Although we have previously noted gingival bleeding in 50% of CHIKV infected cynomolgus monkeys [7], and the presence of blood was noted in some positive samples (Table 2), we have insufficient observations formally to correlate this manifestation with the presence of infectious virus in the saliva of each animal.

### CHIKV in the saliva of CHIKV-infected humans

**Fig 4:**
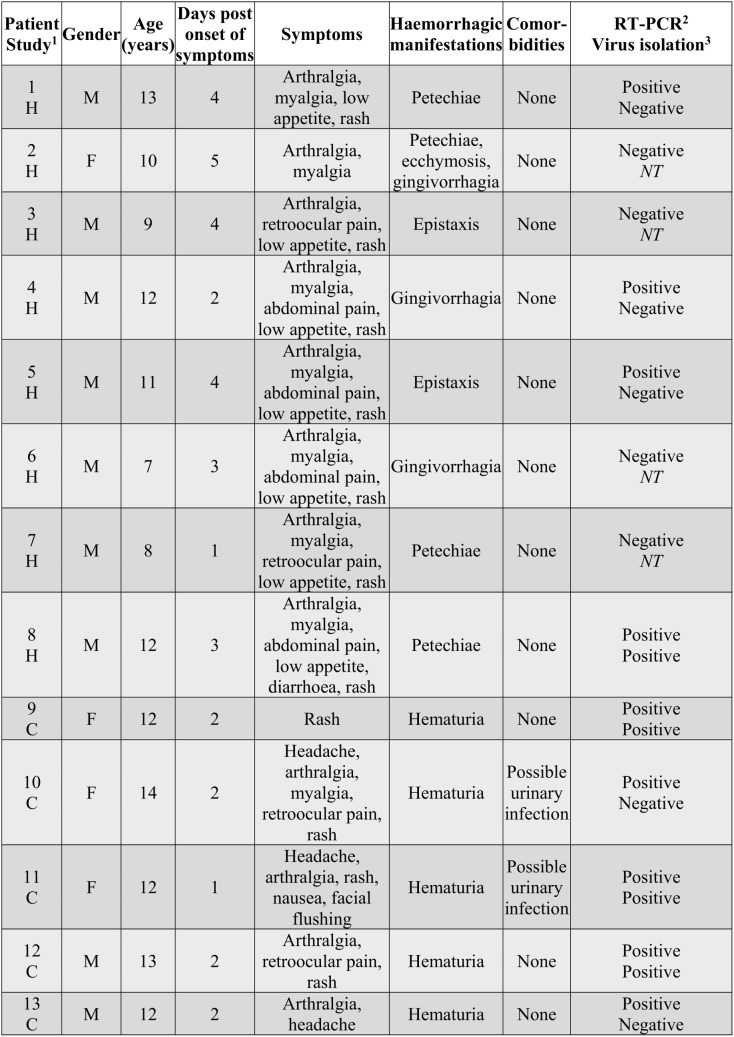
Presence of CHIKV RNA and infectious virus in the saliva of acute CHIKV infected patients. All patients were negative for dengue. Symptoms, manifestations and comorbidities at time of saliva collection. ^1^ H–Hospital study, C–Cohort study. ^2^ CHIKV detected in saliva by TaqMan RT-PCR. ^3^ Saliva was incubated with Vero cells, and TaqMan RT-PCR was used to confirm CHIKV in cultures showing cytopathic effect. *NT*–not tested.

Although hemorrhagic manifestations in CHIKV infected humans are well described [42], and include nose bleeds (epistaxis) [12,16] and gingivorrhagia (bleeding gums) [15,18], the question of whether such manifestations result in the presence of infectious virus in saliva, has to our knowledge, not been addressed. Saliva samples were thus collected from patients enrolled in 2 studies that were initially established to study dengue in Managua, Nicaragua. Saliva samples from 13 acute patients whose blood samples were RT-PCR positive for CHIKV, negative for dengue and who presented with hemorrhagic manifestations, were tested for the presence of CHIKV mRNA by TaqMan real time RT-PCR. CHIKV mRNA was detected in the saliva of 10 of these 13 patients (Fig 4). Saliva samples from 4 of these patients also produced cytopathic effects after culture with Vero cells, with RT-PCR confirming the presence of CHIKV in these cultures. Thus infectious CHIKV can also be present in saliva of CHIKV infected humans.

## Discussion

To our knowledge this study represents the first report showing that oral fluids/saliva from CHIKV infected animals and humans can contain infectious CHIKV. The presence of infectious virus in oral fluids of IRF3/7^-/-^ mice was associated with high viraemia and overt hemorrhagic lesions in the nasal cavities. In cynomolgus monkeys CHIKV was isolated from saliva on or near the peak viraemia, with gingival bleeding noted previously in 50% of infected animals [7]. The presence of infectious CHIKV in the saliva of several acute human patients with hemorrhagic manifestations, provides further support for the contention that CHIKV in saliva is associated with hemorrhagic lesions in the nasal/oral cavities during the viraemic period. The safety implications of infectious saliva are perhaps most relevant for dentistry [18]. Bites from CHIKV infected individuals with hemorrhagic manifestations might also be considered potentially infectious; however, for mice this appears to be restricted to mice deficient in IFNα/β responses [6], with saliva from wild-type mice testing negative.

The blood in the oral cavity of IRF3/7^-/-^ mice appears to arise from hemorrhagic lesions in the olfactory epithelium (Fig 1). Another alphavirus, Venezuelan equine encephalitis virus, efficiently infects the olfactory epithelium following aerosol exposure [43], perhaps suggesting this site is vulnerable to alphavirus infection and disruption. Once the olfactory epithelium is breached, infected blood escaping from blood vessels (e.g. via vasculitis [6]) can reach the nasal, and thus oral, cavities. Whether CHIKV-infected primates develop lesions in the olfactory epithelium is unclear; however, epistaxis (nose bleed) has been reported in humans [12,16] and was also observed in our human cohort. Gingivorrhagia (bleeding gums) was not observed in IRF3/7^-/-^ mice, but has been described in monkeys [7] and humans [15] (and several patients in our cohort) and may also provide a route for virus to enter the oral cavity. Although these hemorrhagic manifestations did not correlate with detection of infectious virus in saliva (Fig 4), they may simply have been inapparent or subclinical at the time of examination.

The ability to show mouse-to-mouse transmission of CHIKV using IRF3/7^-/-^ mice, would appear to be due both to the hemorrhagic manifestations in these mice leading to infectious virus in the oral cavity, and the increased susceptibility of these mice to mucosal infection. Transmission likely arises from transfer of infectious oral fluids (from Donor mice) perhaps taken up orally (by Recipient mice) during grooming and barbering; (the latter two activities are usually contemporaneous). Although CHIKV is usually transmitted by *Aedes aegypti* and *Aedes albopictus* mosquitoes [2], non-vector based transmission of CHIKV has been reported in humans, with mother-to-child infection observed when children are born to viraemic mothers [44]. Perhaps of note, maternal milk (also mucosally-derived) from breast-feeding viraemic women (n = 20) was CHIKV-negative by RT-PCR [44], although hemorrhagic manifestations were not reported for these patients. Non-vector based transmission of a number of other arboviruses via mucosal surfaces has also been reported [45,46], including oral infection of birds with other alphaviruses (Eastern and Western equine encephalitis viruses) [45]. CHIKV has also been reported to be infectious via the intranasal route in wild-type mice [39], with intranasal and aerosol infection with equine encephalitis viruses well described [43,45]. The relevance of non-vector based transmission for enzootic cycles or epidemics is generally likely to be limited, with hemorrhage and deficiencies in IFNα/β responses not common. One might, nevertheless, speculate (given Table 1 and Fig 3) that individuals with a diminished capacity to mount IFNα/β responses might be at increased risk of infection via mucosal surfaces. Such individuals include the elderly [47], neonates [48,49], HIV patients [50], asthmatics [51] and perhaps diabetics [52].

Although the studies herein are hardly exhaustive, the lack of mouse-to-mouse transmission via aerosol (Table 1) is perhaps not surprising. Aerosol transmission of CHIKV has not been formally reported in the publically available literature. Suspected aerosol infections have been reported for CHIKV in humans; however, the circumstances were not described [53]. Two CHIKV infections were reported among a large number of laboratory personnel working in Nigeria (1963–1977); however, the infected staff were living and working in a CHIKV outbreak area [54]. Weaponization may be required for efficient aerosol transmission. CHIKV is classified as a Biosafety Level 3 organism in many jurisdictions (including the USA), in part because of the potential for aerosol infection; an argument perhaps not influential in Singapore where CHIKV is classed as a Biosafety Level 2 organism.

## Supporting Information

S1 File Supporting Information. (a) Survival, (b) foot swelling, (c) viraemia and (d) hemorrhage in IRF3/7^-/-^ mice infected with CHIKV via the i.p. route **(Fig A)**. H&E staining of nasal cavities in C57BL/6 mice day 5 post i.p. infection with CHIKV **(Fig B)**. Graphical representation of the viral RNA data provided in Table 2 for animals C to F **(Fig C)**.
(PDF)
