# Supplementary material for: Infectious Chikungunya Virus in the Saliva of Mice, Monkeys and Humans
Source: PLoS One. 2015 Oct 8;10(10):e0139481. doi: 10.1371/journal.pone.0139481 (PMC4598147; doi:10.1371/journal.pone.0139481)
Supplement: S1 File — (a) Survival, (b) foot swelling, (c) viraemia and (d) hemorrhage in IRF3/7-/- mice infected with CHIKV via the i.p. route (Fig A). H&E staining of nasal cavities in C57BL/6 mice day 5 post i.p. infection with CHIKV (Fig B). Graphical representation of the viral RNA data provided in Table 2 for animals C to F (Fig C). (PDF) [file pone.0139481.s001.pdf]

S1 File

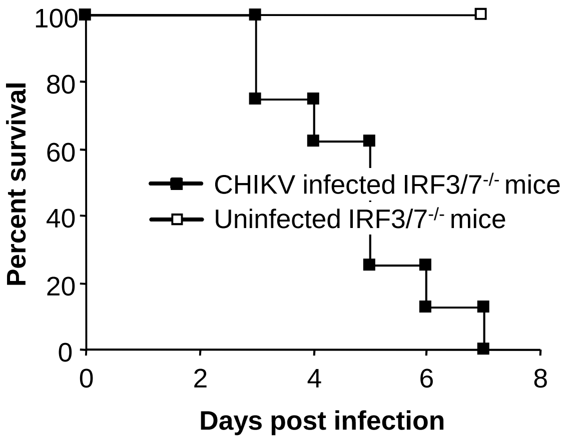

Figure A (a) Survival of IRF3/7$^{-/-}$ mice with and without i.p. inoculation of 2x10^4 $CCID_{50}$ CHIKV. All infected mice showed clinical signs requiring euthanasia within 7 days (n=8). The survival curve is similar to that reported previously for IRF3/7$^{-/-}$ mice infected with the same dose via s.c. injection into the feet (Rudd et al., 2012).

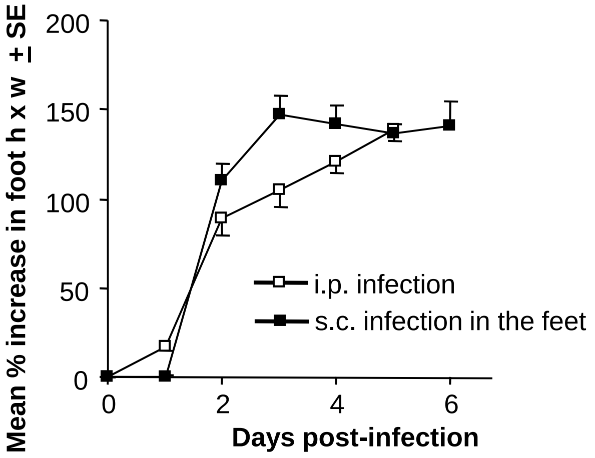

Figure A (b) Foot swelling in IRF3/7$^{-/-}$ mice infected via s.c. injection into the feet (Rudd et al., 2012) or via i.p. inoculation (2x10^4 $CCID_{50}$). Both routes of infection result in significant overt foot swelling (n=3-7 mice per group and time point).

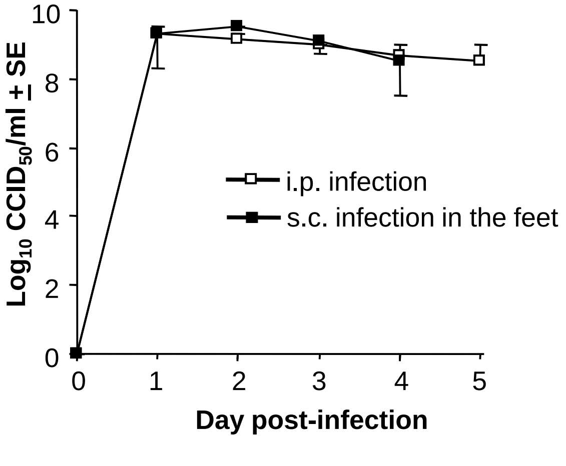

Figure A (c) Viraemia in IRF3/7$^{-/-}$ mice infected via s.c. injection into the feet (Rudd et al., 2012) or via i.p. inoculation (2x10^4 $CCID_{50}$). Both routes of infection result similar viraemia levels (n=3-5 mice per group and time point).

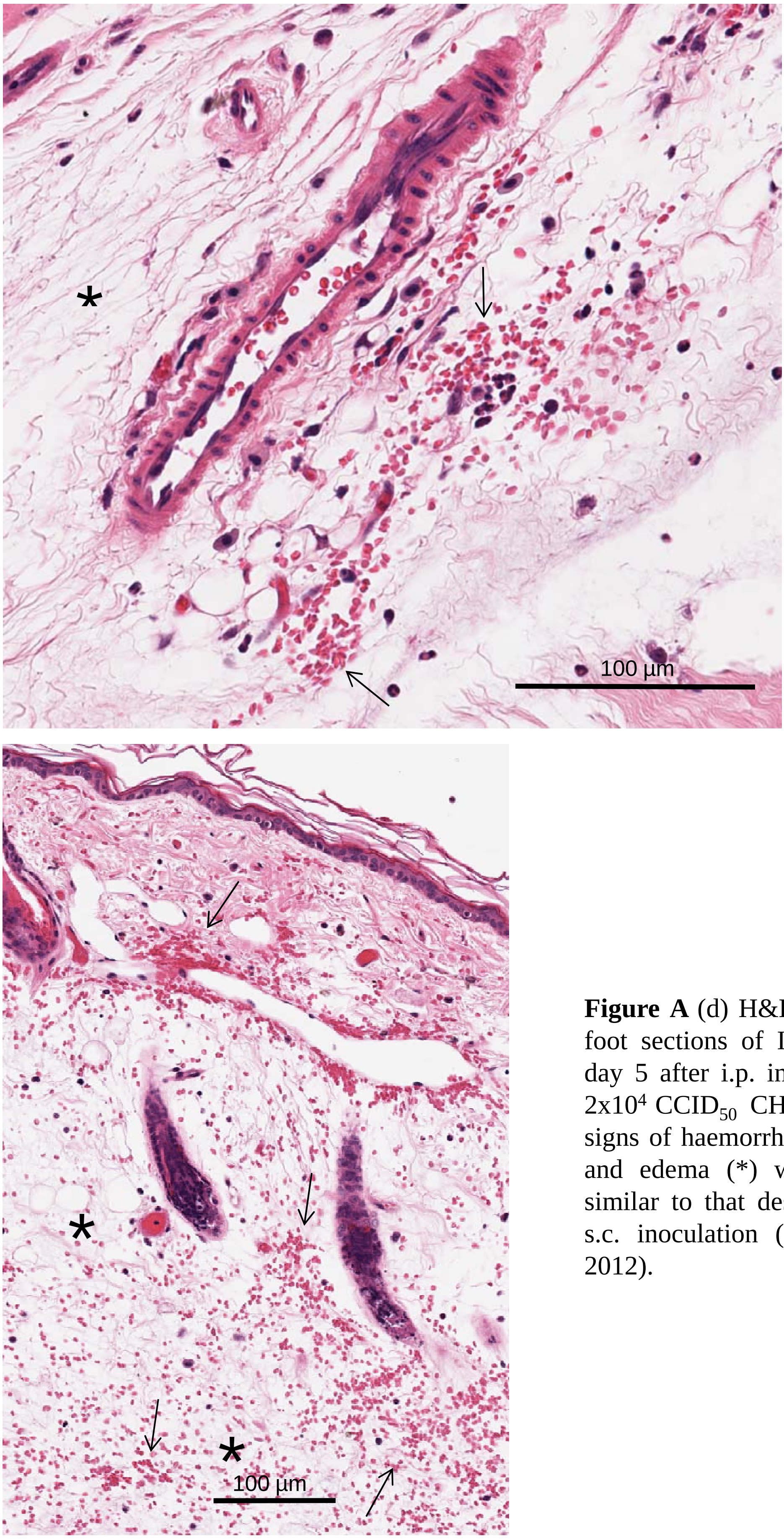

Figure A (d) H&E staining of foot sections of IRF3/7$^{-/-}$mice day 5 after i.p. inoculation of $2x10^4$ $CCID_{50}$ CHIKV. Clear signs of haemorrhage (arrows) and edema (*) were evident similar to that described after s.c. inoculation (Rudd et al. 2012).

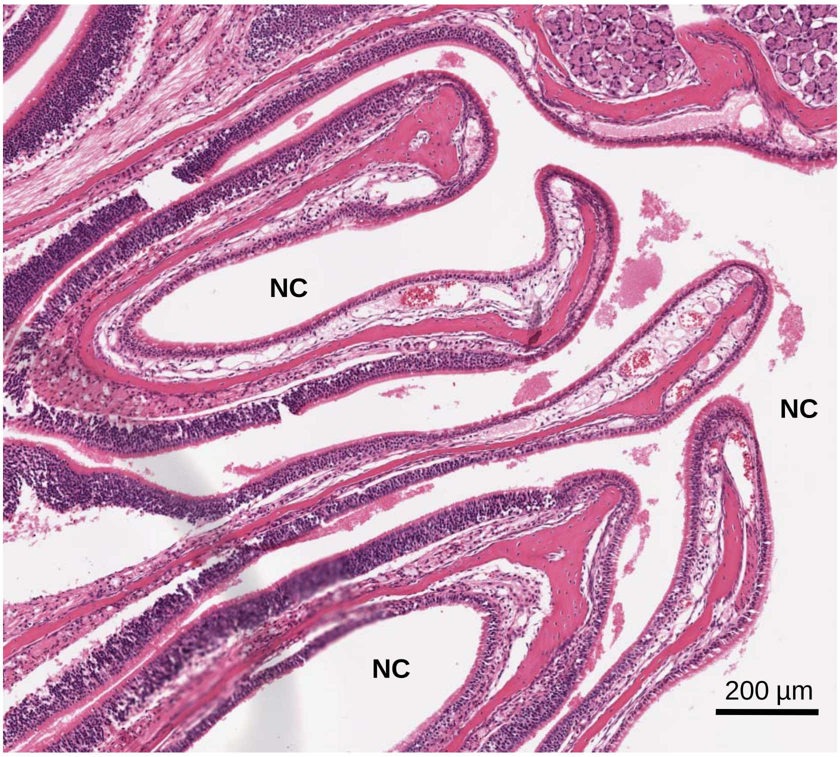

Figure B. H&E of head sections showing nasal cavities of C57BL/6 mice day 5 post i.p. inoculation of CHIKV ($2x10^4$ $CCID_{50}$). No overt hemorrhagic epithelial lesions were found. Occasionally (2 instances in one mouse out of 3 examined) a small number of red blood cells were seen in the nasal cavity (bottom image, arrows). NC – nasal cavity.

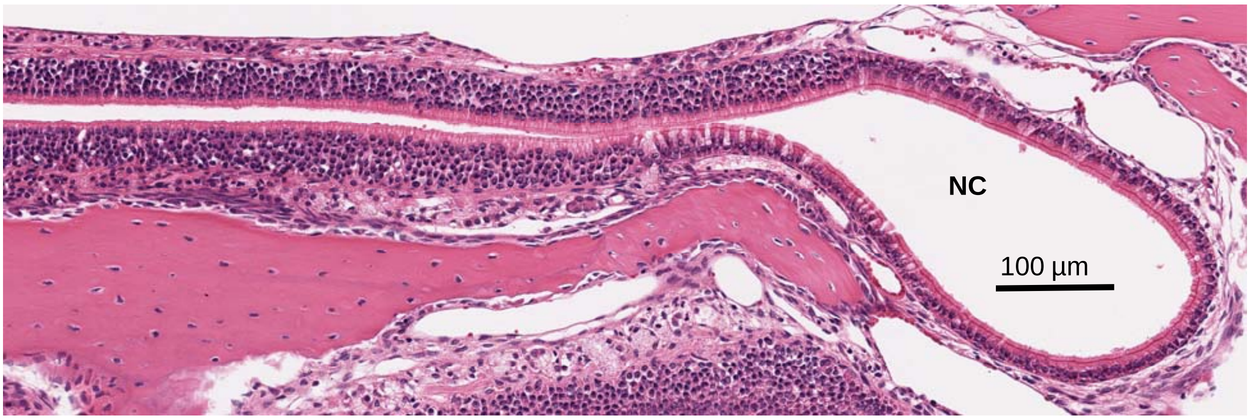

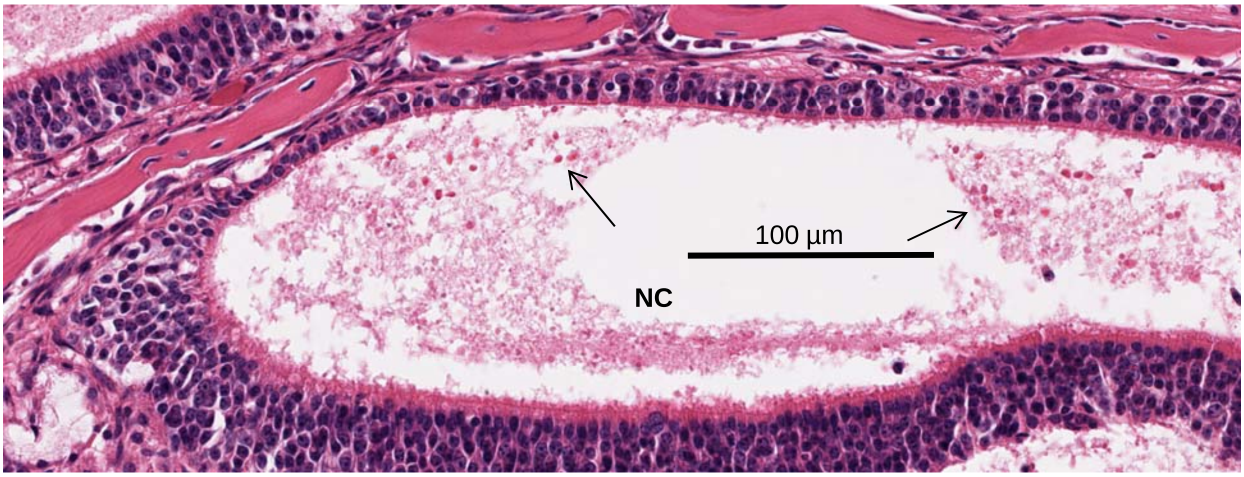

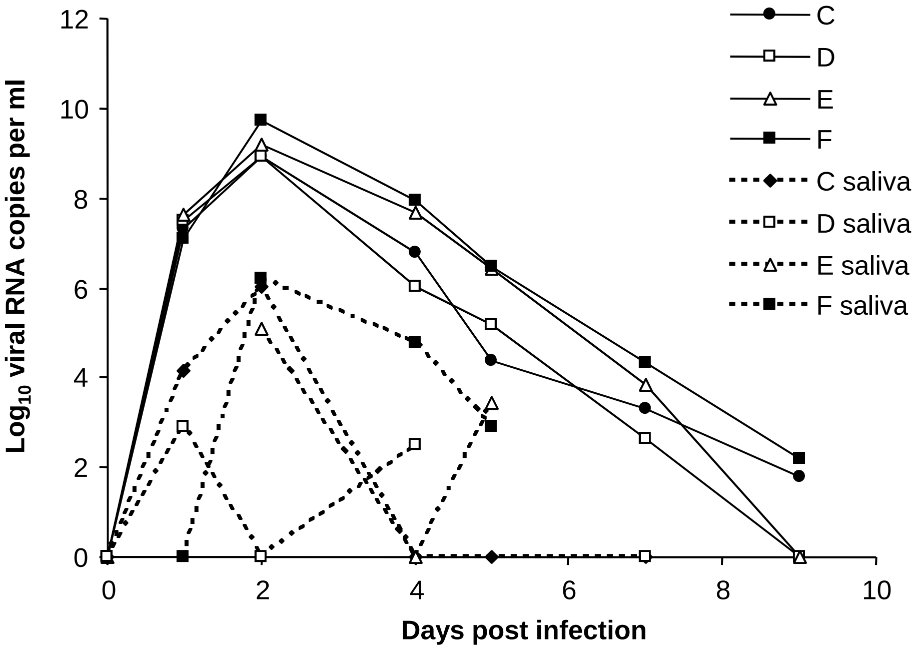

Figure C. Graphical representation of the viral RNA data provided in Table 3 for animals C to F.
